# A novel extracorporeal cardiopulmonary resuscitation strategy using a hybrid emergency room for patients with pulseless electrical activity

**DOI:** 10.1186/s13049-022-01024-2

**Published:** 2022-05-31

**Authors:** Shinichi Ijuin, Akihiko Inoue, Satoshi Ishihara, Masafumi Suga, Takeshi Nishimura, Shota Kikuta, Haruki Nakayama, Nobuaki Igarashi, Shigenari Matsuyama, Tomofumi Doi, Shinichi Nakayama

**Affiliations:** 1 Department of Emergency and Critical Care Medicine, Hyogo Emergency Medical Center, 1-3-1 Wakinohamakaigandori, Chuo-ku, Kobe, 651-0073 Japan; 2 Department of Cardiology, Japanese Red Cross Kobe Hospital, 1-3-1 Wakinohamakaigandori, Chuo-ku, Kobe, 651-0073 Japan

**Keywords:** Extracorporeal cardiopulmonary resuscitation, Hybrid emergency room, Pulseless electrical activity, Pulmonary embolism, Aortic disease; Intracranial haemorrhage

## Abstract

**Background:**

Whether extracorporeal cardiopulmonary resuscitation (ECPR) is indicated for patients with pulseless electrical activity (PEA) remains unclear. Pulmonary embolism with PEA is a good candidate for ECPR; however, PEA can sometimes include an aortic disease and intracranial haemorrhage, with extremely poor neurological outcomes, and can thus not be used as a suitable candidate. We began employing an ECPR strategy that utilised a hybrid emergency room (ER) to perform computed tomography (CT) before extracorporeal membrane oxygenation (ECMO) induction from January 2020. Therefore, the present study aimed to evaluate the effectiveness of this ECPR strategy.

**Methods:**

Medical records of patients who transferred to our hybrid ER and required ECPR for PEA between January 2020 and November 2021 were reviewed.

**Results:**

Twelve consecutive patients (median age, 67 [range, 57–73] years) with PEA requiring ECPR were identified in our hybrid ER. Among these patients, nine were diagnosed using an initial CT scan (intracranial haemorrhage (3); cardiac tamponade due to aortic dissection (3); aortic rupture (2); and cardiac rupture (1)), and unnecessary ECMO was avoided. The remaining three patients underwent ECPR, and two of them survived with favourable neurological outcomes. Patients not indicated for ECPR were excluded before ECMO induction.

**Conclusion:**

Our ECPR strategy that involved the utilisation of a hybrid ER may be useful for the exclusion of patients with PEA not indicated for ECPR and decision making.

## Background

Extracorporeal cardiopulmonary resuscitation (ECPR) is a method used to stabilise hemodynamics and provide end-organ perfusion in cases of inadequate conventional CPR and reversible cause of cardiac arrest (CA). A favourable neurological outcome, defined as a score of 1–2 on the Glasgow-Pittsburgh Cerebral Performance Category scale, is likely achieved by ECPR for patients with CA having a shockable rhythm, which is ventricular fibrillation (VF) or pulseless ventricular tachycardia on the initial electrocardiogram (ECG) [1–3]. Therefore, previous studies on ECPR chose shockable rhythm as the initial rhythm; the indication of ECPR for pulseless electrical activity (PEA) otherwise remains unclear. The clinical challenge is how to identify patients with PEA.

Several studies have reported successful resuscitation by inducing ECPR in patients with PEA [4–6]. Among patients with CA having PEA, pulmonary embolism is a good indication for ECPR [7–10]; however, PEA can sometimes include aortic disease and intracranial haemorrhage [11, 12], which have extremely poor clinical outcomes among patients [13–15], and it can thus not be utilised as a candidate for ECPR. Therefore, these diseases should be identified before the induction of extracorporeal membrane oxygenation (ECMO). However, diagnosis of such diseases is challenging without imaging evaluation.

The advent of interventional radiology-computed tomography (IVR-CT) has considerably affected the management of trauma patients, enabling diagnosis and intervention without simultaneously transferring the patient. The hybrid emergency room (ER) is defined as an integrated system that includes the ER, emergency CT room, IVR room and operating room [16]. Therefore, patients can be transferred from the ambulance directly and examined in the same space without being transferred to other beds. The use of a hybrid ER reportedly decreases mortality in patients with severe trauma by reducing the time until whole-body CT imaging and allowing early initiation of definitive therapy [16, 17]. Furthermore, a hybrid ER reportedly allows safer and rapid cannulation and ECMO initiation [10, 18], in addition to the exclusion of patients not indicated for ECPR before ECMO induction.

We launched a novel ECPR strategy using a hybrid ER to perform CT before ECMO induction to exclude patients with out-of-hospital cardiac arrest (OHCA) not indicated for ECPR from January 2020. Therefore, this study aimed to assess the effectiveness of our ECPR strategy.

## Methods

### Study design and setting

This retrospective descriptive study was performed at Hyogo Emergency Medical Center, a tertiary emergency medical centre, in Japan. Patients are brought to our institution based on the judgement of emergency medical services (EMS). The medical records of patients with OHCA who were identified from the database were reviewed, and their clinical features and relevant data were collected. This study was approved by the ethical committee of Hyogo Emergency Medical Center (approval number: 2020008). Written informed consent for using patients’ data was obtained from their families at the time of admission by the attending physician.

**Fig. 1:**
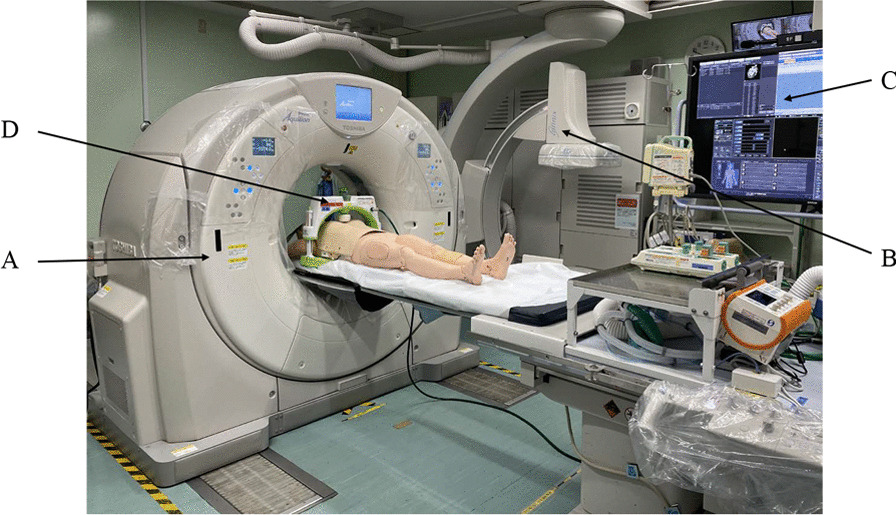
This photograph shows our hybrid ER and the scene while performing CT under mechanical CPR. **A** Sliding gantry computed tomography scanner, **B** movable C-arm, **C** monitoring screen, **D** automatic chest compression device. *ER* Emergency room, *CT* Computed tomography, *CPR* Cardiopulmonary resuscitation

A hybrid ER was installed at our ER in January 2017 and is equipped with a multi-slice IVR-CT system (Aquilion PRIME, TSX-303A; Canon Medical Systems Corp, Tochigi, Japan). Our hybrid ER has a carbon-fibre fluoroscopic table with a movable C-arm combined with a sliding gantry CT scanner (Fig. 1), equipment that allows us to perform diagnostic and therapeutic procedures simultaneously in a single space. At our institution, all trauma patients are taken to the hybrid ER whenever it is available. Medical patients displaying unstable vital signs, indicated for ECPR who have PEA, or with intracranial diseases, such as brain infarction or haemorrhage, are also taken to this room.

### Patients

Patients with OHCA who were transferred to the hybrid ER at Hyogo Emergency Medical Center between January 2020 and November 2021 and required ECPR for PEA were included in this study. In our institution, ECPR is indicated for the following patients: those in the age group of 16–75 years, those whose initial ECG rhythm was shockable regardless of witnessed arrest, those whose initial ECG rhythm was PEA with witnessed arrest, and those whose time from CA to hospital arrival was within 45 min. ECPR was defined as the induction of venoarterial extracorporeal membrane oxygenation (VA-ECMO) in patients with OHCA who did not exhibit the return of spontaneous circulation on hospital arrival. Patients with VF/VT are directly transferred to the angiography room and ECMO induction is performed immediately.

### Procedure

**Fig. 2:**
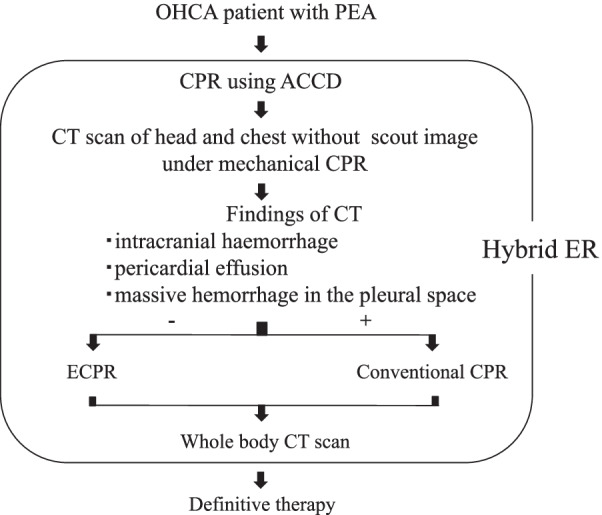
The schema of our protocol in selecting ECPR candidates with PEA. *PEA* Pulseless electrical activity, *CPR* Cardiopulmonary resuscitation, *ACCD* Automatic chest compression device, *CT* Computed tomography, *ECPR* extracorporeal cardiopulmonary resuscitation

Patients with PEA who were candidates for ECPR were directly transferred to the hybrid ER. Mechanical CPR was continuously performed using an automatic chest compression device (ACCD) (CLOVER3000; KOHKEN Medical, Tokyo, Japan). Next, to shorten the time, head and chest CT scans were immediately conducted without scout images under mechanical CPR (Fig. 1). If CT findings did not detect intracranial haemorrhage, pericardial effusion or massive haemorrhage in the pleural space, VA-ECMO was initiated. However, if these findings were indeed detected, conventional CPR was performed without ECMO induction because the patient is strongly expected to have unfavourable neurological outcomes (Fig. 2). The implementation of ECPR was finally decided by the emergency physician. All ECMO cannulations were performed by the emergency physician and cardiologist. The cannulas were inserted into the femoral artery and vein under ultrasonographic and fluoroscopic guidance. We used 17-Fr cannulas for the femoral artery and 21-Fr cannulas for the femoral vein. As necessary, an additional 4-Fr catheter was inserted into the superficial femoral artery to prevent limb ischaemia. The final position of the cannulas and catheters was confirmed by fluoroscopy. If acute coronary syndrome (ACS) is suspected as the cause of CA, coronary angiography is performed after ECMO induction and percutaneous coronary intervention, if necessary.

### Data collection

Data were retrieved from EMS personnel reports, and in-hospital data were retrieved from medical records. We collected data on age, sex, shock delivery from the arrival of EMS to the establishment of ECMO support, time course, CA aetiology and survival and neurological outcomes at hospital discharge. Neurological outcomes were assessed using the Glasgow-Pittsburgh Cerebral Performance Category scale at hospital discharge [1]. Continuous variables are presented as median (interquartile range [IQR]).

## Results

**Fig. 3:**
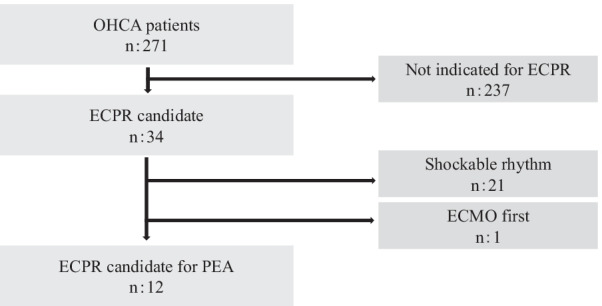
Flowchart of study patients. *OHCA* Out-of-hospital cardiac arrest, *ECPR* extracorporeal cardiopulmonary resuscitation, *ECMO* extracorporeal membrane oxygenation

During the study period, a total of 271 patients with OHCA were transferred to our institute. Among them, 34 patients were ECPR candidates, and 22 of them were excluded from this study because 21 patients had an initial ECG pattern with a shockable rhythm and ECMO induction was performed in 1 patient before conducting a CT scan because of the physician’s decision. Finally, the remaining 12 patients were included in this study (Fig. 3).

**Fig. 4:**
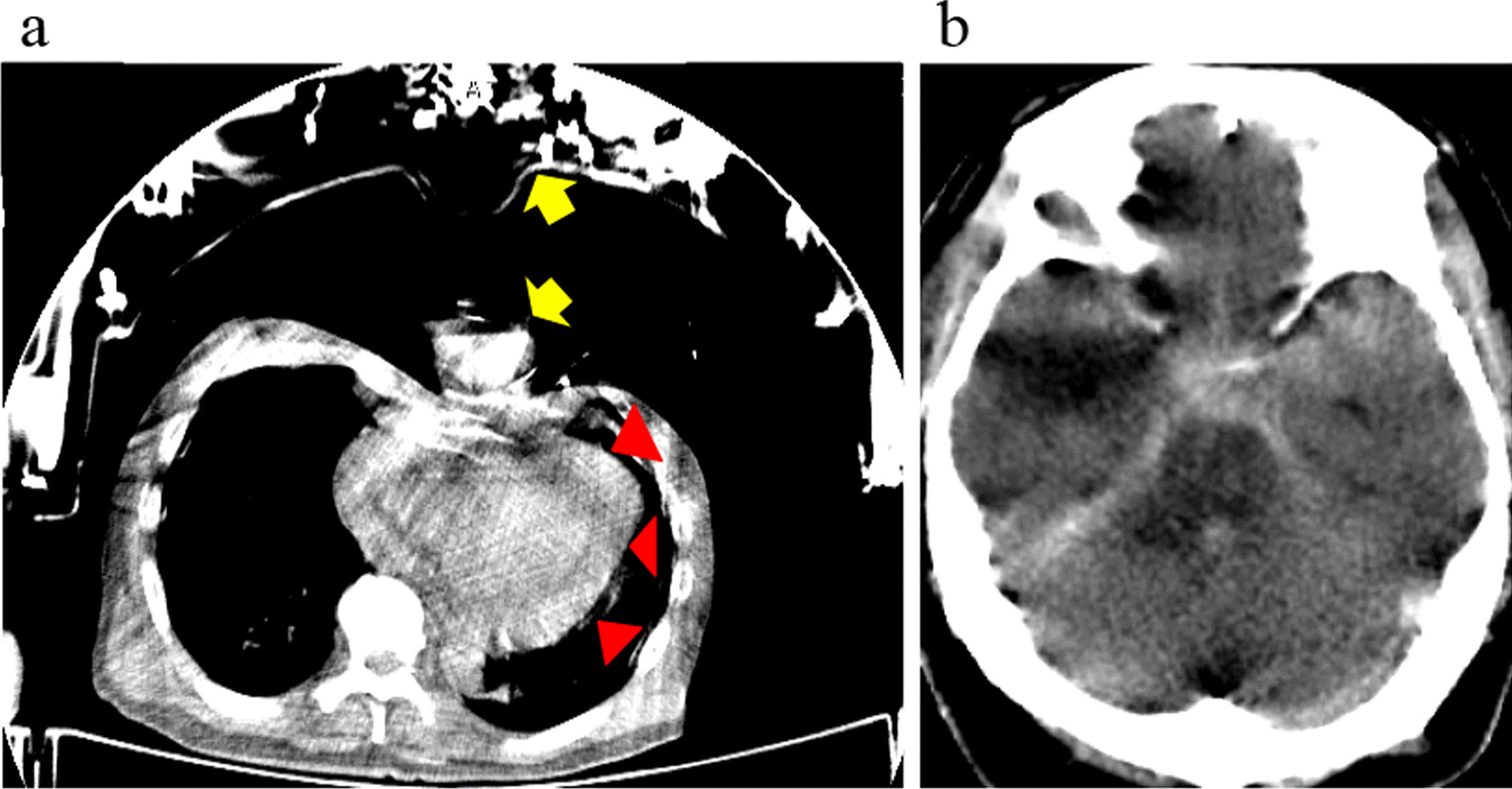
CT scan under mechanical CPR. **a** The CT scan shows pericardial effusion (triangle) and ACCD (arrows) (Case 5). **b** The CT scan shows subarachnoid haemorrhage (Case 7). *CT* Computed tomography, *CPR* Cardiopulmonary resuscitation, *ACCD* Automatic chest compression device

The median age of patients was 67 (IQR, 57–73) years and nine were male. The time from collapse to hospital arrival and the time from arrival to the end of CT were 22 (17–28) minutes and 170 (160–188) seconds, respectively (Table 1). Among them, nine patients were diagnosed using CT without a scout image (intracranial haemorrhage (3); cardiac tamponade due to acute aortic dissection (AAD) (3); aortic rupture (2); and cardiac rupture (1)) (Fig. 4). The remaining three patients underwent ECPR because they did not exhibit any findings associated with intracranial haemorrhage, pericardial effusion or massive haemorrhage in the pleural space by CT scan without a scout image (Case 3, 6 and 8). They were diagnosed with ACS due to occlusion of the left main trunk, aortic dissection complicated with right coronary artery malperfusion without pericardial effusion and pulmonary embolism. No complications associated with ECMO induction were observed. Two (Case 6 and 8) of these patients survived with favourable neurological outcomes. In Case 6, because no findings of cardiac tamponade or rupture were observed on the CT scan regardless of AAD, this patient had favourable outcomes. One patient (Case 3) died on day 12 due to low output syndrome caused by ACS. All patients not indicated for ECPR were excluded before ECMO induction, and unnecessary ECMO was avoided.

**Table 1:** Characteristics and clinical course in OHCA patients with ECPR candidate for PEA

Case	Age/sex	Time from hospital arrival to the end of CT (s)	Diagnosis	ECPR	Low-flow time (min)*	Exclusion before ECMO induction	Outcome	CPC scale
1	57/F	160	TBI (ASDH, Herniation)	No	–	Yes	Death	5
2	74/M	180	Cardiac rupture	No	–	Yes	Death	5
3	80/M	165	ACS (LMT)	Yes	40	Yes	Death	5
4	71/M	160	AAA rupture	No	–	Yes	Death	5
5	69/M	180	AAD-A, Cardiac tamponade	No	–	Yes	Death	5
6	73/M	158	AAD-A, Malperfusion of RCA	Yes	33	Yes	Survival	2
7	38/M	265	SAH	No	–	Yes	Death	5
8	66/M	160	Pulmonary embolism	Yes	21	Yes	Survival	1
9	43/M	248	AAD-A, Cardiac tamponade	No	–	Yes	Death	5
10	58/M	176	TAA rupture	No	–	Yes	Death	5
11	73/F	213	AAD-A,	No	–	Yes	Death	5
12	60/F	158	Cardiac tamponade SAH	No	–	Yes	Death	5

*OHCA* Out-of-hospital cardiac arrest, *CT* Computed tomography, *ECPR* Extracorporeal cardiopulmonary resuscitation, *ECMO* Extracorporeal cardiopulmonary membrane, *CPC* Cerebral Performance Category, *TBI* Traumatic brain injury, *ASDH* Acute subdural haemorrhage, *ACS* Acute coronary syndrome, *LMT* Left main trunk, *AAA* Acute abdominal aneurysm, *AAD-A* Acute aortic dissection Stanford type A, *RCA* Right coronary artery, *SAH* Subarachnoid haemorrhage, *TAA* Thoracic aortic aneurysm

^*^Low-flow time was defined as duration of cardiac arrest or predicted cardiac arrest to the establishment of ECMO support onset

Survival and neurological outcomes were assessed at hospital discharge

## Discussion

In this study, we evaluated the efficacy of ECPR for patients with PEA in a hybrid ER setup. Twelve patients with PEA were transferred as ECPR candidates and were assessed as to whether they were indicated for ECPR based on our hybrid ER strategy. Nine patients did not undergo unnecessary ECMO induction, and two of three patients who underwent ECPR had favourable neurological outcomes. At present, 14 centres in Japan have a hybrid ER, but only our centre has introduced this strategy as far as we know.

Several studies have reported successful resuscitation using ECPR induction for patients with PEA [4–6, 19]. Diek et al. reported that the survival rate of patients with PEA for ECPR was 23.8% [20]. Because shockable rhythms are a good candidate for ECPR [2, 3], non-shockable rhythms may also be a candidate for ECPR [21]. Patients with PEA with witnesses were more likely to have a fatal pulmonary embolism [7], and favourable outcomes were expected by inducing ECMO [8, 9]. Therefore, it seems that CA due to pulmonary embolism is expected to also have a favourable neurological outcome by ECPR induction. Furthermore, some reports demonstrated the use of ECPR in cases of accidental hypothermia with PEA [22, 23]. The disease can be diagnosed based on medical history and physical findings, and aggressive ECMO induction may result in improved neurological outcomes.

Those with poor neurological outcomes after ECPR due to aortic disease and intracranial haemorrhage were not indicated for ECPR [13]. Although ECPR for intracranial haemorrhage has not been reported, CA caused by this condition has been reported to have unfavourable neurological outcomes [14, 15]. These patients could not be considered for ECPR. Therefore, whether ECPR is indicated before ECMO induction should be determined. However, in our study, one patient with AAD without cardiac tamponade and rupture had a favourable neurological outcome (Case 6). Regardless of CA caused by AAD, patients without cardiac tamponade and rupture, for instance, coronary artery malperfusion, may be a candidate for ECPR.

In conventional ECPR performed in the ER or angiography room, these diseases cannot be diagnosed before ECMO induction. A hybrid ER enables a diagnosis to be made on the basis of a CT scan before ECMO induction and simultaneous ECMO induction without relocating the patient. Furthermore, the use of fluoroscopy may be beneficial in terms of avoiding incorrect cannula placement and bleeding complications [18]. Therefore, our protocol is more useful than conventional ECPR. In our study, all patients with diseases not indicated for ECPR were excluded because of the initial CT scan and to avoid unnecessary ECMO induction in hybrid ER. Therefore, our strategy would contribute to the cost-effectiveness of ECPR.

Of the three patients who underwent ECPR, one patient (Case 3) died because of ACS. In this case, it was extremely difficult to detect coronary artery disease as a cause of CA on the initial CT scan before ECMO induction because there were no findings of coronary artery disease such as severe calcification of the coronary artery. The aim of our study was to exclude non-indication of ECPR for patients such as intracranial haemorrhage and aortic diseases. Therefore, we believe that this case was a limitation of this strategy.

Among all the patients in this study, nine patients had a medical history and five of these had a history of cardiac disease. However, because the number of cases is extremely small, it could not be analysed statistically. In situations of ECPR needed, we are often required to make a decision with limited information about medical history.

There are several limitations to this study. First, it was a retrospective, single-centre study. Second, the number of patients may have been small with no other control groups and the investigation was based on a case series targeting a relatively limited number of patients. Third, the responsibility of decision-making regarding the treatment to be conducted was that of the physician.

## Conclusions

Our novel ECPR strategy using hybrid ER may contribute to the diagnosis of patients not indicated for ECPR, such as those with intracranial haemorrhage and aortic disease with cardiac tamponade and rupture before ECMO induction, which may be useful in terms of decision making regarding ECPR. However, information on hybrid ERs is limited and the number of patients is small in the literature; therefore, further research on the efficacy and feasibility of hybrid ERs is necessary.

## Data Availability

The dataset used and analysed during the current study is available from the corresponding author on reasonable request.

## Abbreviations

AAD: Acute aortic dissection
ACCD: Automatic chest compression device
ACS: Acute coronary syndrome
CA: Cardiac arrest
CT: Computed tomography
ECG: Electrocardiogram
ECMO: Extracorporeal membrane oxygenation
ECPR: Extracorporeal cardiopulmonary resuscitation
EMS: Emergency medical services
Hybrid ER: Hybrid emergency room
IQR: Interquartile range
IVR-CT: Interventional radiology-computed tomography
OHCA: Out-of-hospital cardiac arrest
PEA: Pulseless electrical activity
VA-ECMO: Venoarterial extracorporeal membrane oxygenation
VF: Ventricular fibrillation
